# Exploring the Causal Relationship Between Inflammatory Cytokines and MRI‐Derived Brain Iron: A Mendelian Randomization Study

**DOI:** 10.1002/brb3.70181

**Published:** 2024-12-06

**Authors:** Zhounan Wu, Wantong Xu, Xuemei Wang, Dan Peng, Zhongbiao Jiang

**Affiliations:** ^1^ Department of Orthopaedic Surgery The Second Xiangya Hospital, Central South University Changsha Hunan China; ^2^ Department of Laboratory Medicine The Second Xiangya Hospital, Central South University Changsha Hunan China; ^3^ Department of Radiology The Second Xiangya Hospital, Central South University Changsha Hunan China

**Keywords:** brain iron, cytokines, GWAS, inflammation, Mendelian randomization

## Abstract

**Background:**

The association between inflammation and brain iron deposition is widely acknowledged. However, the precise causal impact of peripheral inflammatory cytokines on changes in brain iron content remains uncertain.

**Methods:**

The study utilized an available genome‐wide association study (GWAS) summary associated with inflammatory cytokines from The Cardiovascular Risk in Young Finns Study and the FINRISK surveys. The GWAS data for brain iron markers were obtained from the UK Biobank. We assessed the iron content of each brain region using susceptibility‐weighted magnetic resonance imaging, utilizing both quantitative susceptibility mapping and T2* measurements. The primary outcomes were susceptibility (*χ*) and T2*, which serve as indices of iron deposition. To investigate the causal relationship between exposure and outcome, we primarily employed inverse variance weighting, MR Egger, weighted median, simple mode, and weighted mode methods, collectively enhancing the robustness of our results.

**Results:**

The results of MR analyses demonstrate that our study unveiled that nerve growth factor‐β, hepatocyte growth factor, interleukin‐1 (IL‐1), IL‐8, macrophage inflammatory protein 1α, and tumor necrosis factor‐α were associated with elevated brain iron content in the regions of left hippocampus, putamen, left thalamus, right pallidum, right hippocampus, left amygdala, respectively. Furthermore, our investigation provides evidence for a negative relationship between IL‐1, IL‐17, monocyte chemotactic protein‐3, tumor necrosis factor‐β, and brain iron content in distinct regions.

**Conclusions:**

Our findings suggest a causal association between circulating inflammatory cytokines and brain iron deposition across various brain regions. This provides new insights into the immunopathogenesis of neurodegenerative diseases and potential preventive strategies targeting iron metabolism.

## Graphical abstract

We explored the causal relationship between inflammatory cytokines and MRI‐derived brain iron using a Mendelian randomization approach, based on publicly available genome‐wide association studies (GWAS) data. As a result, brain iron deposition may emerge as a potential mechanism underlying neuroinflammation‐mediated cognitive decline.

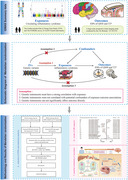

**Abbreviations**
BNGF: nerve growth factor‐β
CNS: central nervous system
CSF‐1R: colony stimulating factor‐1 receptor
GWAS: genome‐wide association study
HGF: hepatocyte growth factor
IDPs: image‐derived phenotypes
IL‐1: interleukin‐1
IL‐17: interleukin‐17
IL‐2: interleukin‐2
IL‐8: interleukin‐8
IVW: inverse variance weighted
MCP3: monocyte chemotactic protein‐3
MIP1α: macrophage inflammatory protein 1α
MR: Mendelian randomization
MS: multiple sclerosis
PD: Parkinson's disease
QC: quality control
QSM: quantitative susceptibility mapping
SNP: single‐nucleotide polymorphism
swMRI: susceptibility‐weighted MRI
TNF‐α: tumor necrosis factor‐α
TNF‐β: tumor necrosis factor‐β

## 1 Introduction

Iron plays a vital role in various brain physiological processes, including serving as a cofactor in myelin formation, neurotransmission, oxygen transport, cell division, and mitochondrial energy production (Thirupathi and Chang 2019). However, imbalances in iron metabolism can lead to brain dysfunction and damage (Ficiarà, Stura, and Guiot 2022), and higher iron levels or deficiencies potentially impair normal brain cell function (D'Mello and Kindy 2020; Parks and Wharton 1989). The accumulation of various iron complexes in brain regions linked to motor and cognitive impairment is observed during the aging process (Ward et al. 2014). Research has revealed an association between excessive iron accumulation and multiple neurodegenerative diseases common in the elderly population, including Friedreich ataxia and Alzheimer's disease (AD) (Hagemeier, Geurts, and Zivadinov 2012; Zecca et al. 2004). In addition, dysregulated iron metabolism plays a critical role in the progression of central nervous system (CNS) tumors such as glioblastoma, contributing to tumor growth and therapeutic resistance (Caverzan and Ibarra 2024). Consequently, maintaining precise regulation of brain iron metabolism is crucial, making the study of iron metabolism in the CNS an increasingly intriguing area of research.

While the specific pathophysiology of brain iron deposition, a common pathological brain change, remains elusive, numerous previous studies have substantiated the critical role of inflammation in its onset and progression (Rosenblum and Kosman 2022). Many observational studies have identified the coexistence of neuroinflammation and brain iron deposition in patients with various neurodegenerative diseases, including Parkinson's disease (PD) and Huntington's disease (Pajares et al. 2020; Subhramanyam et al. 2019). In addition, an animal study has suggested a significant link between brain iron accumulation in specific regions of murine brain tissue and markers of brain inflammation during natural aging (Ellison et al. 2022). It has been demonstrated that both proinflammatory and anti‐inflammatory stimuli can alter the iron uptake patterns of brain cells such as microglia, astrocytes, and other neural cells, resulting in disrupted iron metabolism and subsequent secondary neuroinflammation (McCarthy et al. 2018; Ward, Dexter, and Crichton 2022). Under the induction of neuroinflammation, the permeability of the blood‐brain barrier increases, allowing both monocytes loaded with iron and noncellular iron to migrate into the CNS, while chronic inflammation and the oxidative stress it induces further promote iron deposition (Andersen, Johnsen, and Moos 2014). Due to the CNS's slow iron turnover, iron accumulates in large quantities in brain cells, and excess iron, in turn, amplifies inflammatory responses, oxidative stress, and lipid peroxidation, ultimately leading to neuronal death and brain dysfunction (Li et al. 2024). Many inflammatory cytokines play critical roles in this process. Increases in interleukin‐6 (IL‐6) levels, whether in the systemic circulation or within the brain, have been shown to stimulate the production of hepcidin, a molecule that binds to ferroportin, promoting intracellular iron accumulation (Wessling‐Resnick 2010). In addition, tumor necrosis factor‐α (TNF‐α) has the capacity to induce iron ccumulation in cells independently of hepcidin‐induced iron buildup by stimulating the expression of divalent metal transporter 1 (DMT1) (Sharma et al. 2005). At present, the role of inflammation in the occurrence and development of various CNS disorders is becoming more and more clear, making immunotherapy regimen (especially immune molecular‐targeted therapies) a very promising therapeutic option. An animal study demonstrated that modulation of peripheral immune homeostasis in AD through the peripheral infusion of low‐dose recombinant human IL‐2 reduced neuroinflammation, significantly decreased Aβ plaque deposition, and slowed cognitive decline in experimental mice at mid‐disease stages (Yuan et al. 2023). Colony stimulating factor‐1 receptor (CSF‐1R) inhibitors have also demonstrated beneficial effects in preclinical models of neurodegenerative diseases (Han et al. 2022). Therefore, clarifying the role of circulating inflammatory cytokines in brain iron deposition will enhance our understanding of how iron metabolism imbalances and ferroptosis contribute to the development of various inflammation‐associated neurological disorders, and may also help in identifying potential targets for preventive immunotherapy.

However, despite prior research unveiling associations between inflammation and iron deposition, the question of whether a causal relationship exists between inflammatory cytokines and brain iron deposition remains a subject of debate. Several observational research have attempted to clarify this connection, yet results from these studies may be affected by the unexpected confounding variables and reverse causality, which complicates the determination of a definitive causal association. Recently, Mendelian randomization (MR) has gained favor as an epidemiological approach for causal inference, utilizing genetic variation as an instrumental variable (IV). Since the distribution of genotypes passed from parent to child at conception is random, the confounding factors do not affect the relationship between genetic variation and outcome. This random allocation of genotypes minimizes confounding, thereby providing a more reliable basis for inferring causality. MR analyses are anchored on the following assumptions: (1) the IVs are strongly correlated with the exposure variables, (2) the IVs are not subject to any confounders, and (3) the effect of the IVs on the outcome is mediated exclusively through the exposure and are not influenced by other pathways.

A growing body of research points to an association between inflammatory cytokines and brain iron deposition. However, no previous study has explored the effects of inflammatory cytokines on iron content in specific brain regions using MR. Therefore, this study pioneers a two‐sample MR approach, using relevant genome‐wide association study (GWAS) data from a large European population, to examine the causal relationship between peripheral inflammatory cytokines and MRI‐derived brain iron.

## 2 Materials and Methods

### 2.1 Data Sources for Inflammatory Cytokines

Regarding the exposure data, we used GWAS data on 41 peripheral cytokines and utilized them to quantify the levels of circulating inflammatory cytokines. This GWAS data are derived from a cohort study involving 8293 Finnish individuals, which amalgamated findings from two primary sources: The Cardiovascular Risk in Young Finns Study (YFS) and the FINRISK survey (Ahola‐Olli et al. 2017). The YFS is a multicenter follow‐up investigation involving randomly selected participants from the Finnish cities of Helsinki, Kuopio, Oulu, Tampere, and Turku, along with their surrounding areas. The YFS study includes 2019 unrelated individuals in the 2007 follow‐up and 1664 unrelated individuals in the 2011 follow‐up, all of whom had cytokine genotype data measured. The FINRISK survey, on the other hand, is a population‐based cross‐sectional study conducted at 5‐year intervals to assess the prevalence of risk factors for chronic diseases in Finland. Each survey involves the random selection of individuals aged 25–74 from five different geographical regions across Finland. The FINRISK study utilized cytokine genome‐wide data from 4608 subjects who participated in 1997 and 1705 subjects who participated in 2002. The average age of participants in the YFS survey was 37 years, while the average age of participants in the FINRISK survey was 60 years.

### 2.2 Data Sources for image‐derived phenotypes of Quantitative Susceptibility Mapping and T2*

The GWAS data on brain iron‐related imaging phenotypes used in this study were derived from a large‐scale prospective brain imaging study conducted by the UK Biobank that aimed to utilize the collected brain imaging data to explore the potential of medical imaging for early disease prediction (Miller et al. 2016). To capture a wide range of imaging phenotypes associated with underlying brain diseases, the study incorporated six imaging protocols: T1‐weighted, T2‐weighted, susceptibility‐weighted MRI (swMRI), diffusion MRI, and both task and resting‐state functional MRI. Among these, the signal of swMRI is influenced by substances whose magnetic susceptibility differs significantly from that of tissue water, such as iron, myelin, and calcium. To assess the brain iron deposition in various regions, we relied on quantitative susceptibility mapping (QSM) and T2* measurements, both of which were derived from the swMRI data (Wang et al. 2022). These two distinct yet complementary metrics, QSM and T2*, were used to generate image‐derived phenotypes (IDPs), whose primary outcomes were susceptibility (*χ*) and T2*, respectively, as indicators of iron deposition. For QSM generation, phase images collected from individual coil channels underwent combination, masking, and unwrapping. Utilizing a QSM pipeline involving background field removal, dipole inversion, and CSF referencing, *χ* was computed (Wang et al. 2022). The calculation of the inverse of T2* was performed using magnitude images derived from two echo times. After noise minimization via spatial filtering of T2* images, a linear registration transformed them into the T1 space. The median T2* value was then estimated individually for each subcortical structure regions of interest acquired from the T1 (Alfaro‐Almagro et al. 2018). This dual approach compensates for the respective limitations of each method, enhances the sensitivity in detecting brain iron deposition levels, and provides stronger support for our conclusions (Zheng et al. 2012; McCrea et al. 2008). The raw data for QSM and T2* were obtained from the 35,885 participants in the previously mentioned UK Biobank Brain Imaging study, published in early 2020, all of whom underwent swMRI scans. Notably, all participants were perfectly healthy at the time of their scan participation. A total of 53.11% were females, aged 45–82 years (64.04 ± 7.5 years) at imaging time. In addition, the study designers implemented a rigorous quality control (QC) pipeline to ensure the reliability of the imaging data. During the scans, specific data were collected to monitor the data quality, with participants being excluded if they exhibited excessive head motion, atypical structures, or anatomical abnormalities. Full details of the QC pipeline have been described in previous studies (Alfaro‐Almagro et al. 2018). After excluding data that did not meet the QC pipeline, the swMRI results of the remaining 35,273 participants were developed for the data of IDPs. Among them, 29,579 unrelated participants with recent UK ancestry were selected for GWAS data to minimize confounding effects related to environmental or demographic factors. All relevant data from the UK Biobank have been centrally processed, and GWAS data of IDPs for QSM and T2* are now available for analysts to utilize directly. The selection of ethnically diverse cohort studies as data sources for both the exposure and outcome groups helps exclude potential effects arising from population overlap.

### 2.3 Instrumental Variable

First, we set a genome‐wide significance threshold of *p* < 5 × 10^−6^ to identify single‐nucleotide polymorphisms (SNPs) that were highly correlated with inflammatory cytokines and IDPs of QSM and T2*. These carefully chosen SNPs were designated as potential IVs for subsequent MR analysis. To avoid weak instrument bias, we calculated *F*‐statistics, a recognized metric of instrument robustness. Generally, instruments with an *F*‐statistic below 10 were excluded, as they are typically regarded as “weak instruments” (Deng et al. 2020). We clumped these SNPs (*r* ^2^ < 0.001, window size = 10,000 kb), computed their *F*‐statistics, and manually excluded SNPs associated with potential confounding factors.

### 2.4 Statistical Analysis

In this study, we utilized a comprehensive suite of MR techniques to investigate the causal relationships between inflammatory cytokines and IDPs of QSM and T2*. These techniques included inverse variance weighting (IVW), MR‐Egger regression, weighted median, simple mode and weighted mode. In addition, sensitivity analyses were conducted to address potential pleiotropy bias, enhancing the reliability of our results.

Given its reputation for reliability, we selected the IVW approach as the primary method for evaluating causal effects (Burgess, Butterworth, and Thompson 2013). Furthermore, we employed five complementary MR techniques to strengthen the robustness of our results. In the sensitivity analysis, we calculated Cochran's *Q* and Rucker's *Q* test (*p* < 0.05) to assess heterogeneity for IVW and MR‐Egger methods, respectively. In addition, we utilized the MR‐PRESSO global test and calculated MR‐Egger's intercept to identify potential pleiotropy, setting a significance threshold of *p* < 0.05. After observing horizontal pleiotropy, outlier SNPs identified by MR‐PRESSO were excluded, and MR estimates were recalculated to assess. Since a total of 28 IDP data of QSM and T2* were included, a *p* value of 1.79E^−03^ (0.05/28) was set to make the results more rigorous.

All analyses were conducted using the two‐sample MR (version 0.5.6) and MR‐PRESSO (version 1.0) packages within R version 4.2.1 (Hemani et al. 2018; Verbanck et al. 2018).

## 3 Result

**FIGURE 1:**
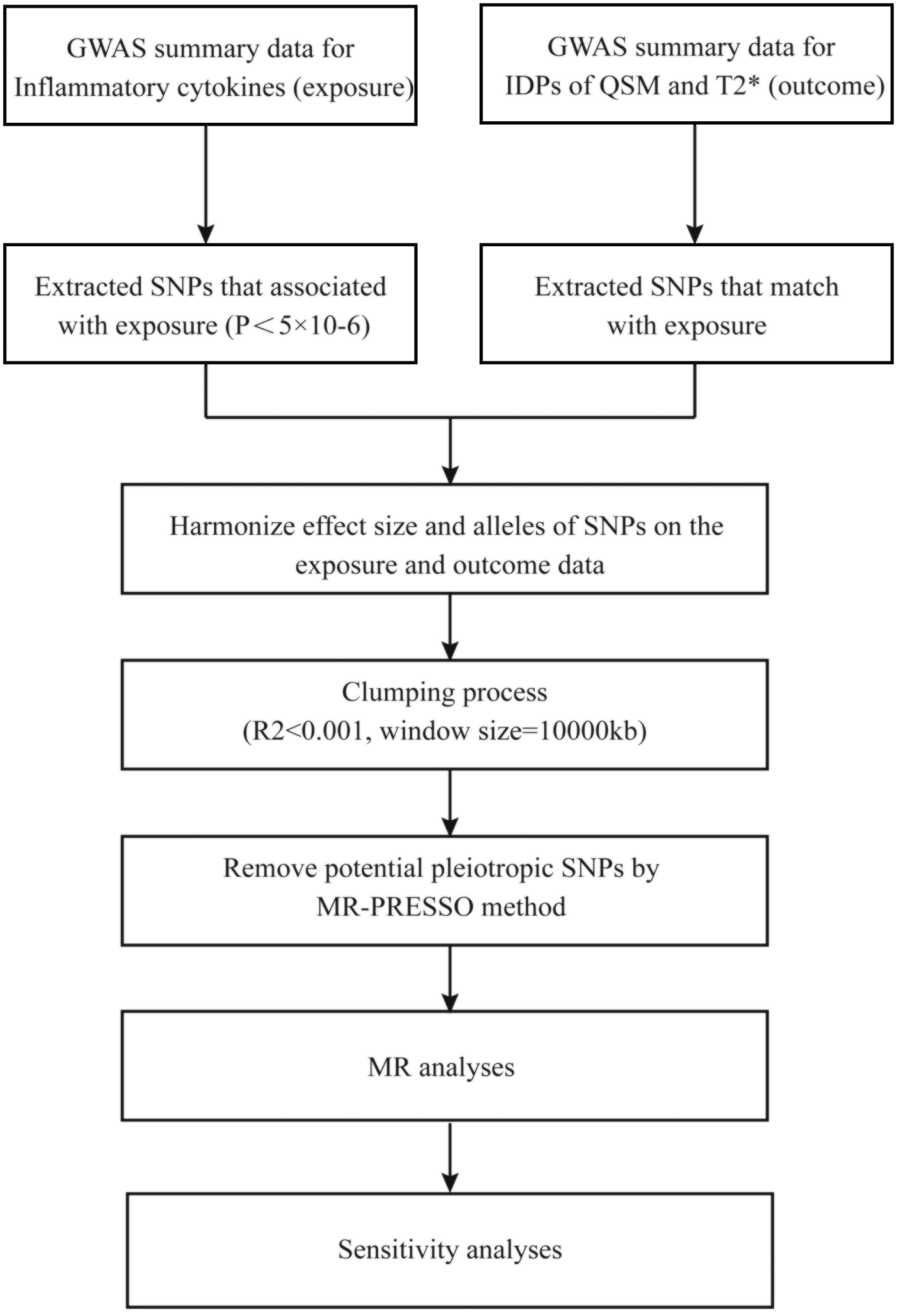
Schematic of the study design in this Mendelian randomization (MR) analysis. Significant instrumental variables were selected for 41 inflammatory cytokines and QSM and T2*, and the causal relationship was then explored.

Figure 1 succinctly illustrates the workflow of our MR analysis. Initially, we identified 14,269 SNPs associated with 41 inflammatory cytokines using a suggestive significance threshold of *p* < 5.0 × 10^−6^. Specific IVs for each category of inflammatory cytokines are detailed in Table S4. Table S2 provides a comprehensive compilation of SNPs associated with iron deposition in various brain structures after meticulous harmonization and clumping. We can then guarantee the absence of horizontal pleiotropy among the remaining IVs by excluding pleiotropic SNPs identified through the MR‐PRESSO outlier test (*p* > 0.05).

**FIGURE 2:**
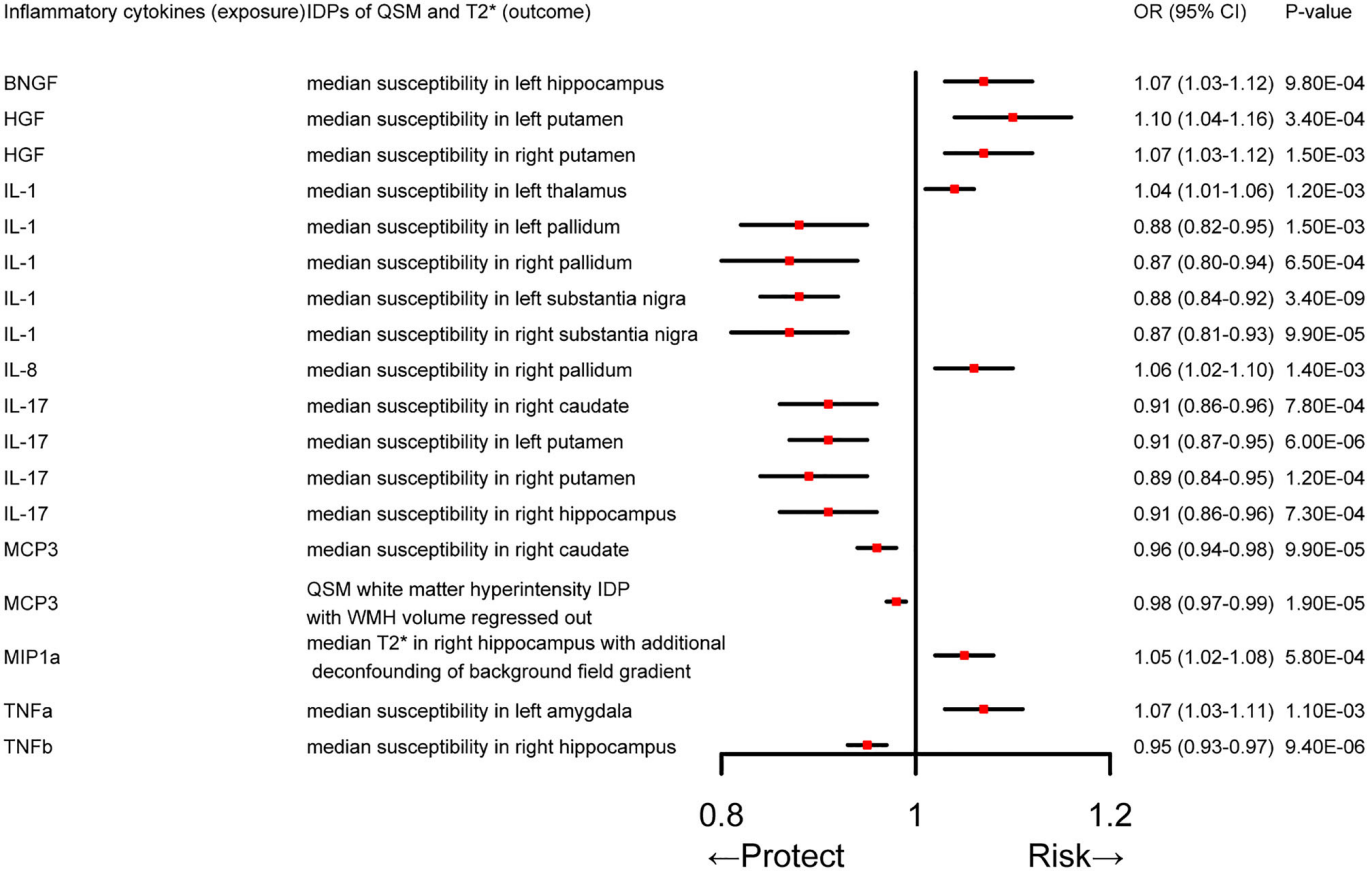
Causal correlations of 41 inflammatory cytokines on IDPs of QSM and T2*. The change in the odds ratio (OR) of IDPs of QSM and T2* per one‐SD rise in the cytokine level is shown by OR and 95% confidence interval. *p* value of 1.79E−03 was found significant after multiple‐comparison correction. The results from inverse variance weighted method were shown for all cytokines, which was considered as the recommended method.

In the MR analysis, we employ five different approaches to explore the causal relationship between each category of inflammatory cytokines and IDPs of QSM and T2* in various regions of brain tissue. As seen in Figure 2, the results of IVW analyses demonstrate that hepatocyte growth factor (HGF) was positively correlated with susceptibility in both left (OR = 1.10, 95% CI: 1.04–1.16, *p*_IVW_  = 3.40E^−04^) and right (OR = 1.07, 95% CI: 1.03–1.12, *p*_IVW_  = 1.50E^−03^) putamen. IL‐1 was positively correlated with the left thalamus (OR = 1.04, 95% CI: 1.01–1.06, *p*_IVW_  = 1.20E^−03^). Furthermore, it was observed that nerve growth factor‐β (BNGF), IL‐8, and TNF‐α exhibited positive correlations with susceptibility in the left hippocampus (OR = 1.07, 95% CI: 1.03–1.12, *p*_IVW_ = 9.80E^−04^), right pallidum (OR = 1.06, 95% CI: 1.02–1.10, *p*_IVW_ = 1.40E^−03^), and left amygdala (OR = 1.07, 95% CI: 1.03–1.11, *p*_IVW_ = 1.10E^−03^), respectively. Macrophage inflammatory protein 1α (MIP1α) showed a positive correlation with T2* in the right hippocampus with additional deconfounding of background field gradient (OR = 1.05, 95% CI: 1.02–1.08, *p*_IVW_ = 5.80E^−04^) at the genetic level. In addition to the above positive correlation, some cytokines were negatively correlated with IDPs of QSM and T2* of different brain regions. IL‐1 was negatively correlated with susceptibility in the following four regions: left pallidum (OR = 0.88, 95% CI: 0.82–0.95, *p*_IVW_ = 1.50E^−03^), right pallidum (OR = 0.87, 95% CI: 0.80–0.94, *p*_IVW_ = 6.50E^−04^), left substantia nigra (OR = 0.88, 95% CI: 0.84–0.92, *p*_IVW_ = 3.40E^−09^), and right substantia nigra (OR = 0.87, 95% CI: 0.81–0.93, *p*_IVW_ = 9.90E^−05^). IL‐17 was negatively correlated with susceptibility in three regions: left putamen (OR = 0.91, 95% CI: 0.87–0.95, *p*_IVW_ = 6.00E^−06^), right putamen (OR = 0.89, 95% CI: 0.84–0.95, *p*_IVW_ = 1.20E^−04^), right hippocampus (OR = 0.91, 95% CI: 0.86–0.96, *p*_IVW_ = 7.30E^−04^); whereas MCP3 was negatively correlated with the susceptibility in right caudate (OR = 0.96, 95% CI: 0.94–0.98, *p*_IVW_ = 9.90E^−05^), and had a negative effect on the QSM white matter hyperintensity IDP with WMH volume regressed out (OR = 0.98, 95% CI: 0.97–0.99, *p*_IVW_ = 1.90E^−05^). In addition, TNF‐β displayed a negative correlation with susceptibility in the right hippocampus (OR = 0.95, 95% CI: 0.93–0.97, *p*_IVW_ = 9.40E^−06^). As shown in Table 1, the assessment results from other supplementary MR methods were directionally consistent with those from the IVW method. The MR results were obtained by taking the intersection of the Tables S1.1 and S1.2.

**TABLE 1:** Full positive results of MR estimate for the association between inflammatory cytokines and IDPs of QSM and T2*.

Inflammatory cytokines (exposure)	IDPs of QSM and T2* (outcome)	MR method	No. SNP	*F*	OR (95% CI)	*p* value
BNGF	Median susceptibility in left hippocampus	IVW(MRE)	4	35.45	1.07 (1.03–1.12)	9.80E−04
		MR Egger	4		1.09 (0.67–1.75)	0.77
		Weighted median	4		1.08 (0.98–1.18)	0.14
		Simple mode	4		1.03 (0.90–1.18)	0.67
		Weighted mode	4		1.10 (0.97–1.25)	0.23
HGF	Median susceptibility in left putamen	IVW(MRE)	9	21.24	1.10 (1.04–1.16)	3.40E−04
		MR Egger	9		1.08 (0.93–1.27)	0.34
		Weighted median	9		1.11 (1.01–1.22)	2.30E−02
		Simple mode	9		1.09 (0.97–1.23)	0.18
		Weighted mode	9		1.11 (1.00–1.23)	0.09
HGF	Median susceptibility in right putamen	IVW(MRE)	9	21.24	1.07 (1.03–1.12)	1.50E−03
		MR Egger	9		1.06 (0.90–1.24)	0.51
		Weighted median	9		1.09 (0.99–1.19)	0.08
		Simple mode	9		1.08 (0.96–1.22)	0.25
		Weighted mode	9		1.08 (0.98–1.20)	0.16
IL‐1	Median susceptibility in left thalamus	IVW(MRE)	4	25.55	1.04 (1.01–1.06)	1.20E−03
		MR Egger	4		1.04 (0.84–1.30)	0.74
		Weighted median	4		1.05 (0.96–1.15)	0.32
		Simple mode	4		1.05 (0.93–1.18)	0.5
		Weighted mode	4		1.05 (0.93–1.18)	0.49
IL‐1	Median susceptibility in left pallidum	IVW(MRE)	4	25.55	0.88 (0.82–0.95)	1.50E−03
		MR Egger	4		0.82 (0.64–1.03)	0.23
		Weighted median	4		0.90 (0.81–1.00)	4.70E−02
		Simple mode	4		0.91 (0.78–1.05)	0.29
		Weighted mode	4		0.91 (0.78–1.06)	0.31
IL‐1	Median susceptibility in right pallidum	IVW(MRE)	4	25.55	0.87 (0.80–0.94)	6.50E−04
		MR Egger	4		0.88 (0.66–1.16)	0.47
		Weighted median	4		0.87 (0.79–0.97)	1.20E−02
		Simple mode	4		0.85 (0.72–1.01)	0.16
		Weighted mode	4		0.89 (0.76–1.04)	0.24
IL‐1	Median susceptibility in left substantia nigra	IVW(MRE)	4	25.55	0.88 (0.84–0.92)	3.40E−09
		MR Egger	4		0.90 (0.72–1.12)	0.45
		Weighted median	4		0.87 (0.79–0.96)	4.00E−03
		Simple mode	4		0.86 (0.75–0.98)	0.11
		Weighted mode	4		0.86 (0.76–0.97)	0.09
IL‐1	Median susceptibility in right substantia nigra	IVW(MRE)	4	25.55	0.87 (0.81–0.93)	9.90E−05
		MR Egger	4		0.85 (0.67–1.07)	0.3
		Weighted median	4		0.89 (0.80–0.98)	2.40E−02
		Simple mode	4		0.91 (0.77–1.06)	0.31
		Weighted mode	4		0.91 (0.79–1.04)	0.26
IL‐8	Median susceptibility in right pallidum	IVW(MRE)	8	21.11	1.06 (1.02–1.10)	1.40E−03
		MR Egger	8		1.06 (0.99–1.14)	0.15
		Weighted median	8		1.07 (1.01–1.14)	2.50E−02
		Simple mode	8		1.07 (0.98–1.17)	0.16
		Weighted mode	8		1.07 (0.99–1.14)	0.12
IL‐17	Median susceptibility in left putamen	IVW(MRE)	11	22.96	0.91 (0.87–0.95)	6.00E−06
		MR Egger	11		0.86 (0.76–0.98)	0.06
		Weighted median	11		0.89 (0.82–0.98)	1.20E−02
		Simple mode	11		0.90 (0.78–1.03)	0.16
		Weighted mode	11		0.88 (0.77–1.01)	0.1
IL‐17	Median susceptibility in right putamen	IVW(MRE)	11	22.96	0.89 (0.84–0.95)	1.20E−04
		MR Egger	11		0.84 (0.73–0.96)	2.70E−02
		Weighted median	11		0.89 (0.82–0.98)	1.40E−02
		Simple mode	11		0.90 (0.78–1.04)	0.18
		Weighted mode	11		0.85 (0.74–0.97)	3.50E−02
IL‐17	Median susceptibility in right hippocampus	IVW(MRE)	11	22.96	0.91 (0.86–0.96)	7.30E−04
		MR Egger	11		0.83 (0.73–0.94)	2.00E−02
		Weighted median	11		0.90 (0.82–0.99)	2.30E−02
		Simple mode	11		0.95 (0.82–1.08)	0.44
		Weighted mode	11		0.90 (0.80–1.02)	0.13
MCP3	Median susceptibility in right caudate	IVW(MRE)	6	26.44	0.96 (0.94–0.98)	9.90E−05
		MR Egger	6		0.98 (0.88–1.08)	0.65
		Weighted median	6		0.96 (0.91–1.01)	0.12
		Simple mode	6		0.95 (0.88–1.01)	0.17
		Weighted mode	6		0.95 (0.89–1.02)	0.2
MCP3	QSM white matter hyperintensity IDP with WMH volume regressed out	IVW(MRE)	6	26.44	0.98 (0.97–0.99)	1.90E−05
		MR Egger	6		0.98 (0.88–1.08)	0.67
		Weighted median	6		0.99 (0.94–1.03)	0.56
		Simple mode	6		0.99 (0.93–1.05)	0.72
		Weighted mode	6		0.99 (0.93–1.05)	0.69
MIP1α	Median T2* in right hippocampus with additional deconfounding of background field gradient	IVW(MRE)	6	21.4	1.05 (1.02–1.08)	5.80E−04
		MR Egger	6		1.04 (0.85–1.28)	0.7
		Weighted median	6		1.04 (0.95–1.13)	0.4
		Simple mode	6		1.03 (0.91–1.17)	0.63
		Weighted mode	6		1.03 (0.92–1.16)	0.61
TNF‐α	Median susceptibility in left amygdala	IVW(MRE)	5	23.09	1.07 (1.03–1.11)	1.10E−03
		MR Egger	5		1.07 (0.97–1.18)	0.29
		Weighted median	5		1.08 (1.00–1.16)	4.10E−02
		Simple mode	5		1.08 (0.98–1.20)	0.2
		Weighted mode	5		1.08 (0.98–1.20)	0.2
TNF‐β	Median susceptibility in right hippocampus	IVW(MRE)	5	21.83	0.95 (0.93–0.97)	9.40E−06
		MR Egger	5		0.94 (0.90–0.99)	0.11
		Weighted median	5		0.95 (0.91–0.99)	7.20E−03
		Simple mode	5		0.95 (0.89–1.01)	0.2
		Weighted mode	5		0.95 (0.91–0.99)	0.06

Abbreviations: IVW, inverse variance weighted; MRE, multiplicative random effects model; MR Egger, Mendelian randomization‐Egger; OR, odds ratio.

A total of 11 causal effects were established between inflammatory cytokines and IDPs of QSM and T2* across various regions of brain tissue. The *F*‐statistic of the IVs fell within the range of 21.11–35.45, suggesting the absence of any weak IV bias, as detailed in Table 1. The results of both the IVW test and the MR‐Egger method indicated the absence of significant heterogeneity among these IVs. In addition, to assess the potential presence of horizontal pleiotropy, we employed the MR‐Egger intercept and conducted the MR‐PRESSO global test. Notably, all *p* values exceeded 0.05, indicating that there was no significant directional horizontal pleiotropy. These results are summarized in Tables 2, S3, and S5.

**TABLE 2:** Sensitivity analyses for association between inflammatory cytokines and IDPs of QSM and T2*.

Inflammatory cytokines (exposure)	IDPs of QSM and T2* (outcome)	No. SNP	Pleiotropy		Heterogenenity	
			MR‐PRESSO Global *p* value	MR‐Egger *p* value	IVW test *p* value	MR‐Egger *p* value
BNGF	Median susceptibility in left hippocampus	4	0.8558	0.968775756	0.84119181	0.65949026
HGF	Median susceptibility in left putamen	9	0.8628	0.835006566	0.81913933	0.73804867
HGF	Median susceptibility in right putamen	9	0.9553	0.84102367	0.93604478	0.89143787
IL‐1	Median susceptibility in left thalamus	4	0.9718	0.954015116	0.97262238	0.89329768
IL‐1	Median susceptibility in left pallidum	4	0.477	0.560633092	0.41661770	0.31767628
IL‐1	Median susceptibility in right pallidum	4	0.4575	0.911894064	0.35684669	0.20093885
IL‐1	Median susceptibility in left substantia nigra	4	0.859	0.81050361	0.82329093	0.65890229
IL‐1	Median susceptibility in right substantia nigra	4	0.583	0.837576304	0.52103552	0.33348452
IL‐8	Median susceptibility in right pallidum	8	0.8124	0.932672969	0.75982960	0.65453316
IL‐17	Median susceptibility in left putamen	11	0.9242	0.419987108	0.92819369	0.93158114
IL‐17	Median susceptibility in right putamen	11	0.6344	0.31171794	0.60403050	0.62614548
IL‐17	Median susceptibility in right hippocampus	11	0.7265	0.141620698	0.69296234	0.85596023
MCP3	Median susceptibility in right caudate	6	0.9488	0.786719148	0.94648930	0.89436907
MCP3	QSM white matter hyperintensity IDP with WMH volume regressed out	6	0.9994	0.906264407	0.99896173	0.99542149
MIP1α	Median T2* in right hippocampus with additional deconfounding of background field gradient	6	0.9802	0.952637689	0.97710981	0.93914308
TNF‐α	Median susceptibility in left amygdala	5	0.7749	0.998447614	0.78224993	0.62664117
TNF‐β	Median susceptibility in right hippocampus	5	0.8454	0.641996851	0.81079364	0.72356846

Abbreviations: IVW, inverse variance weighted; MR‐PRESSO, Mendelian Randomization Pleiotropy RESidual Sum and Outlier; MR‐Egger, Mendelian randomization‐Egger.

## 4 Discussion

Using MR analysis, we investigated the causal relationship between 41 circulating inflammatory cytokines and IDPs of QSM and T2*. Our study unveiled that BNGF, HGF, IL‐1, IL‐8, MIP1α, and TNF‐α were associated with elevated brain iron content in the regions of left hippocampus, putamen, left thalamus, right pallidum, right hippocampus, left amygdala, respectively. Furthermore, our investigation provides compelling evidence for a negative causal relationship between IL‐1, IL‐17, MCP3, TNF‐β, and brain iron content in distinct regions (IL‐1: pallidum, substantia nigra; IL‐17: putamen, right hippocampus; MCP3: right caudate, white matter; TNF‐β: right hippocampus).

Prior research has corroborated several of our findings. The compromised integrity of the blood‐brain barrier allows peripheral inflammatory cells and cytokines to contribute to neuroinflammation (Abe et al. 2020). Proinflammatory cytokines, including IL‐1β, IL‐6, and others, have the capacity to activate microglia, promoting their M1 (proinflammatory) phenotype, and trigger the production and release of proinflammatory cytokines such as TNF‐α and IL‐6 (Fan, Xie, and Chung 2017). These cytokines, in turn, orchestrate persistent chronic inflammation. Furthermore, it's been established that inflammatory cytokines such as TNF‐α and IL‐6 induce the synthesis of DMT1, fostering iron accumulation in both neurons and microglia (Urrutia, Mena, and Núñez 2014). IL‐6 has been demonstrated to elevate the expression of hepcidin in astrocytes and neurons through STAT3 phosphorylation, ultimately leading to brain iron deposition (Qian et al. 2014; You et al. 2017). In addition, studies have detected elevated levels of proinflammatory mediators—such as IL‐1β, IL‐6, IL‐8, and TNF‐α—in the blood and cerebrospinal fluid of AD patients (Leng and Edison 2021). Collectively, these investigations provide substantial support for the notion that heightened levels of IL‐1, TNF‐α, and IL‐8 may represent potential upstream drivers of brain iron deposition.

**FIGURE 3:**
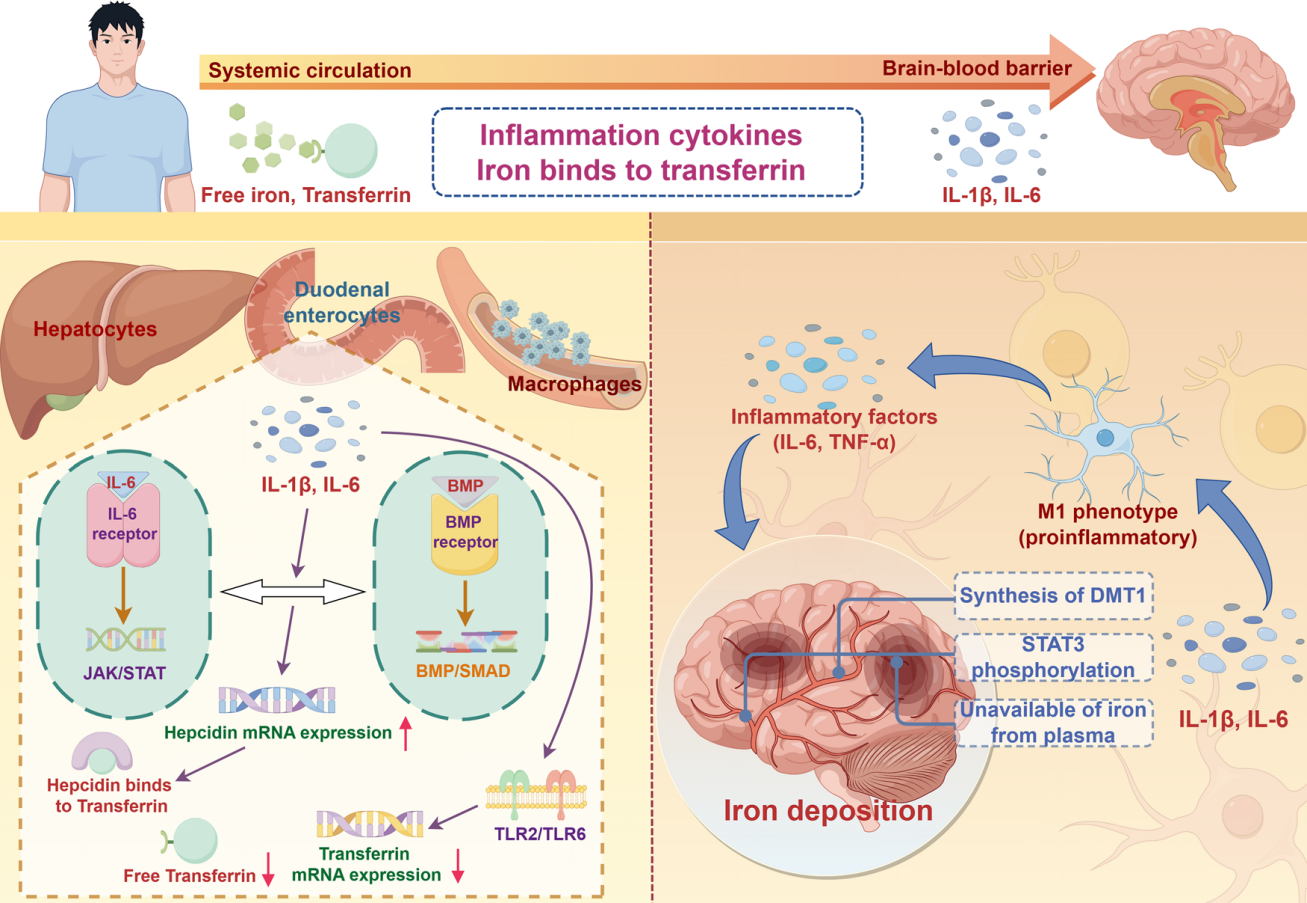
Mechanism of inflammatory cytokines on iron in systemic circulation and brain tissue. During peripheral inflammation, specific cytokines such as IL‐1β and IL‐6 induce a series of signaling molecular interactions (Inamura et al. 2005; Lee et al. 2005) that induce upregulation hepcidin of via the BMP‐SMAD and JAK/STAT pathways (Sangkhae and Nemeth 2017). Simultaneously, inflammatory factors activate TLR2/TLR6, resulting in decreased transcription and production of ferroportin (Guida et al. 2015). Hepcidin binds to ferroportin, obstructing iron efflux by blocking channels and promoting the degradation of iron‐loaded ferroportin (Aschemeyer et al. 2018). This process restricts the export of iron to the plasma, particularly from macrophages, duodenal enterocytes, and hepatocytes (Nemeth and Ganz 2021). Consequently, elevated hepcidin levels and reduced transcription of ferroportin reduce the supply of plasma iron, resulting in functional iron deficiency and unavailability of iron in brain tissue. When the integrity of the blood‐brain barrier is compromised, peripheral inflammatory cells and cytokines can infiltrate brain tissue. These proinflammatory cytokines, including IL‐1β and IL‐6, can activate microglia, promoting their M1 (proinflammatory) phenotype, and trigger the production and release of proinflammatory cytokines such as TNF‐α and IL‐6 (Fan, Xie, and Chung 2017). These cytokines, in turn, orchestrate persistent chronic inflammation. Furthermore, inflammatory cytokines such as TNF‐α and IL‐6 induce the synthesis of DMT1, fostering iron accumulation in both neurons and microglia (Urrutia, Mena, and Núñez 2014). IL‐6 can also elevate the expression of hepcidin in astrocytes and neurons through STAT3 phosphorylation, ultimately leading to brain iron deposition (Qian et al. 2014; You et al. 2017).

The study's findings showed an unexpected negative correlation between common inflammatory mediators including IL‐1, IL‐17, MCP3 and magnetic susceptibility of brain structure, contrary to conventional expectations. We propose this counterintuitive result might be attributed to iron deprivation in erythrocytes and other tissues due to iron sequestration in plasma by circulating inflammatory cytokines (Pasricha et al. 2021), an association currently only observed for select cytokines such as IL‐1β and IL‐6 (Inamura et al. 2005; Lee et al. 2005). Hepcidin binds to ferroportin and blocks iron efflux by blocking channels and inducing the degradation of iron‐loaded ferroportin (Aschemeyer et al. 2018). This process curtails iron export to the plasma, particularly from macrophages, duodenal enterocytes, and hepatocytes (Nemeth and Ganz 2021). During inflammation, a series of signaling molecules interact via the BMP‐SMAD and JAK/STAT pathways to induce the upregulation of hepcidin (Sangkhae and Nemeth 2017). Consequently, elevated hepcidin levels and reduced transcription of ferroportin (Guida et al. 2015) diminish plasma iron supply, resulting in functional iron deficiency and unavailability of iron in brain tissue. Research has demonstrated that iron deficiency can reduce myelin production, impede synaptogenesis, and diminish basal ganglia function, leading to iron‐deficiency cognitive dysfunction, especially in children (Pivina et al. 2019). However, BHGF and HGF have been shown to protect cells from iron‐induced loss (Li et al. 2020; Zhang et al. 1993), and the discrepancy between these previous studies and the results we have derived makes our conclusions need to be explored in further clinical studies. The above potential mechanisms are presented in Figure 3.

In addition, we find it intriguing that IL‐6 and IL‐1β appear in key positions on both sides of Figure 3, suggesting that these two cytokines play critical roles in both inflammation‐mediated iron deposition in the nervous system and inflammation‐driven functional iron deficiency in the peripheral system. The site, type, and nature of inflammation, as well as the ability to disrupt the blood‐brain barrier to allow infiltration of brain tissue by peripherally circulating IL‐6, appear to be key determinants of the effect of IL‐6 on brain iron deposition. However, despite their seemingly central roles, our findings did not reveal an association between genetically predicted IL‐6 and magnetic susceptibility in different brain regions at the SNP level. There are also no GWAS studies demonstrating that IL‐6 can interfere with brain iron by affecting blood iron biomarkers or directly influencing iron metabolism in the brain. This lack of association is not limited to genetic studies. For example, an in vivo and post‐mortem study has demonstrated an association between plasmatic ferritin concentrations and IL‐6 levels in patients with early PD who have a disease duration of below 5 years (Martin‐Bastida et al. 2021). Another observational research from China found that neither any significant difference in peripheral IL‐6 levels between PD patients and controls nor was there a statistical association between IL‐6 concentrations and QSM values in PD patients (Xu et al. 2022). Similarly, an observational study from a European population reported no association between IL‐6 and brain iron content in a cognitively healthy population (Spence et al. 2024). Given these inconsistent findings, further studies are needed to elucidate the exact role of IL‐6 in the progression of brain iron deposition.

Changes in *χ* and T2* serve as potential indicators of variations in both iron content and myelin levels (Duyn and Schenck 2017; Stüber et al. 2014). A notable distinction between *χ* and T2* lies in how they respond to iron (paramagnetic) and myelin (antimagnetic) influences within QSM and T2*, respectively. Specifically, these two factors exert opposing effects on *χ* in QSM but exert the same effect on T2* measurements. Consequently, the positive correlation observed between inflammatory cytokines and *χ* could potentially be driven by an increase in iron content or a decrease in myelin levels. Conversely, the positive correlation between inflammatory cytokines and T2* may result from an increase in either iron content or myelin levels. Several studies have demonstrated the potential of QSM and T2* measurements in diagnosing multiple sclerosis (MS), a progressive demyelinating disease (Wisnieff et al. 2015; Fiscone et al. 2024). In MS, innate immune responses and infiltration of the CNS by abnormally activated immune cells upregulate proinflammatory mediators and activate microglia/macrophages, leading to inflammation and demyelination (Pegoretti et al. 2020). In addition, multiple GWAS studies have demonstrated that genetic susceptibility to MS is associated with a variety of inflammatory factors, including tumor necrosis factor receptor 1, IL‐2, interferon, and nuclear factor kappa‐B (Dendrou, Fugger, and Friese 2015), suggesting that inflammatory cytokines may be potential risk factors for the destruction of myelin. In this study, we noted a negative correlation between MIP1α and T2* in the right hippocampus, suggesting a plausible hypothesis that MIP1α may play a role in mediating the development of demyelinating lesions in the CNS, consequently contributing to an increase in T2* (Janssen et al. 2016). However, given that QSM and T2* cannot definitively identify the underlying etiology of changes in magnetic susceptibility but only provide clues, further research is required to better elucidate the role of inflammatory factors in various demyelinating diseases.

The study boasts several strengths. First, it stands as the first MR investigation to probe the causal link between 41 inflammatory cytokines and brain iron deposition, utilizing the most current data summaries available. Traditional observational studies are often susceptible to bias from reverse causality and confounding, but MR effectively sidesteps reverse causality and minimizes residual confounding, lending greater credibility to the results. Furthermore, we employed multiple alternative methods to gauge the causal relationship between these factors, consistently obtaining effect estimates that corroborate the robustness of our findings. Lastly, unlike previous studies that relied on serum iron or transferrin saturation data (Yuan et al. 2020), our study hinged on imaging data from T2* and QSM, offering a more precise and scientific measurement of brain iron content.

Nonetheless, we must recognize certain limitations of this study. First, this study primarily drew from a population of European origin, which may make it potentially difficult to generalize our conclusions to other populations. Second, the reliability of this MR study depends on the robustness of the original GWAS data, which means the results may remain confined to the genetic level without capturing true causality. Therefore, caution is needed when interpreting these findings, and further clinical studies are crucial to validate and refine these relationships. Third, this study did not screen for specific indicators due to data limitations and could only provide a preliminary description of their causal relationship, which requires more research to further refine. Finally, we must acknowledge that the SNP phenotypes of both inflammatory cytokines and brain MRI imaging data are influenced by factors such as the environment, genetic background, and age of exposure. For example, myelin levels may vary based on lifestyle and environmental factors (Langley et al. 2020; Yoon et al. 2016; Forbes and Gallo 2017). Thus, it is essential to account for these potential biases when interpreting the genetic findings.

## 5 Conclusion

Our study has uncovered a causal relationship between several inflammatory cytokines and IDPs of QSM and T2*, which may serve as indicators of brain iron deposition. Consequently, brain iron deposition may emerge as a potential mechanism underlying neuroinflammation‐mediated cognitive decline. This provides new insights into the immunopathogenesis of neurodegenerative diseases and potential preventive strategies targeting iron metabolism. Further studies are warranted to verify the direct causality between inflammatory cytokines and brain iron deposition.

## Author Contributions

**Zhounan Wu**: writing–review and editing, writing–original draft, visualization, methodology, formal analysis. **Wantong Xu**: data curation, visualization, formal analysis, methodology. **Xuemei Wang**: methodology, formal analysis. **Dan Peng**: conceptualization, methodology, software, writing–review and editing. **Zhongbiao Jiang**: conceptualization, funding acquisition, software, writing–review and editing.

## Conflicts of Interest

The authors declare no conflicts of interest.

### Peer Review

The peer review history for this article is available at https://publons.com/publon/10.1002/brb3.70181.

## Supporting information



Table S1.1: Results of the first part of MR estimates for the association between inflammatory cytokines AND IDPs of QSM and T2*
Table S1.2: Results of the second part of MR estimates for the association between inflammatory cytokines AND IDPs of QSM and T2*

Table S2: Detailed information of instrumental variables used in MR analyses

Table S3: Sensitivity analysis of instrumental variables used in MR analyses

Table S4: GWAS summary data of inflammatory cytokines at *p*‐value < 5.0×10^−6^

Table S5: Sensitivity analysis of instrumental variables used in MR analyses.

## Data Availability

The GWAS summary statistics used to perform the analyses described in the study were obtained from publicly available published data. All data generated or analyzed in the study were included in the article and supporting information.
